# Awareness Level of Radiation Protection among Dental Students

**DOI:** 10.31729/jnma.3651

**Published:** 2018-08-31

**Authors:** Deepanshu Garg, Deepika Kapoor

**Affiliations:** 1 Department of Oral Medicine and Radiology, College of Medical Sciences and Teaching Hospital, Bharatpur, Nepal; 2 Department of Pedodontics, College of Medical Sciences and Teaching Hospital, Bharatpur, Nepal

**Keywords:** *awareness*, *dental*, *radiation*, *students*

## Abstract

**Introduction:**

Dental radiographs have always been the mainstay of the diagnosis of oral diseases. Even if the radiation hazard posed by the dental radiographs is low but still accumulation of such low level radiation in human body over a time period can pose a threat to the overall health of the individual. The aim of this study is to assess the knowledge of dental students about radiation protection and hazards.

**Methods:**

The present cross sectional study was conducted by enrolling one hundred 4^th^ year dental students from the two teaching hospitals in Chitwan district of Nepal. A questionnaire consisting of a total of 20 questions was distributed and each question was allocated 0.5 marks for correct response whereas no deduction for wrong answer.

**Results:**

The results of the present study depicted that 85 (85%) of the total students enrolled in the study had “good” knowledge about the radiation physics, protection and hazards whereas only 15 (15%) of them had “poor” knowledge.

**Conclusions:**

The study concluded that teachers should involve in imparting more and more knowledge as well as training about the topic and dental curriculum should be altered so as to train the budding dentists about all aspects of radiation.

## INTRODUCTION

Awareness about their oral health is increasing day by day among the people all over the globe. Earlier people used to ignore their oral health but with increasing demands to get their oral condition diagnosed, the use of dental x-rays is also increasing.

The study was done because these days the concerns regarding the accumulation of radiation in human body have been growing which can be due to inadvertent use of radiation to take x-rays, use of x-ray machines which have become older leading to leakage of x-rays and more x-rays, invention of cone beam computed tomography and inadequate knowledge of x-rays and their side effects among dental students, dentists, radiographers and other health care workers involved in taking the x-rays.^1^

The aim of this study is to assess the knowledge of dental students about radiation protection and hazards along with their capability to implement this knowledge so as to practice a radiation hazard free dentistry.

## METHODS

The present cross sectional descriptive study was conducted by enrolling one hundred 4th year dental students including those who were present at the time of study from the two teaching hospitals in Chitwan district of Nepal (convenience sampling). The study was initiated in May 2018 with approval of proposal to COMSTH-IRC and it was completed with the preparation of manuscript in the starting of month of June 2018. A questionnaire was distributed to all the dental students of 4th year who were willing to participate. The questionnaire consisted of questions involving socio demographic data, any previous exposure to radiation protection lectures and courses, radiation biology, radiation protection and questions regarding safe radiation protection practice.

The questionnaire consisted of 20 questions and each question was allocated 0.5 marks for correct response whereas no deduction for wrong answer. The knowledge of dental students about the topic was categorized into “poor” and “good” based upon the scores they got after evaluation of their responses. Those who got between 0–5 were considered having “poor” knowledge and those who got marks from 5–10 were considered having “good” knowledge. The results were tabulated in Microsoft Excel and statistical significance was observed knowing the p-value using SPSS software.

## RESULTS

**Figure 1.**
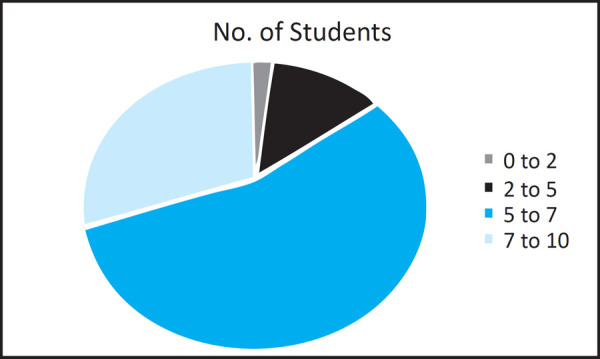
The scores of dental students about the awareness of radiation physics and biology.

The results of the present study depicted that 85 (85%) of the total students enrolled in the study had “good” knowledge about the radiation physics, protection and hazards whereas only 15 (15%) of them had “poor” knowledge. Out of these 85 (85%) students, 55 (55%) of them scored between 5–7 and 30 (30%) of them scored between 7–10 (Figure 1.).

This gives an impression that most of the students are well aware of the concepts of radiation which include radiation physics, protection and hazards along with their implication in safe clinical practice. The results of the study were not significantly different from the results of a previous study done by Enabuele and Igbinedion 2013 which showed 64.1% of total students had “good” knowledge (P = 0.10) about the topic.^8^

## DISCUSSION

These days, dental awareness is increasing among the people globally and people tend to take much more care of their oral cavity these days in contrary to older times.^2^ Earlier people used to neglect their oral health and hygiene but these days due to increased awareness among masses, dental health care has become an important part of the health care system.^3^

As there is increase in awareness, more and more people have started visiting private dental clinics and teaching hospitals for getting an oral health check up and dental radiographs have always been the mainstay of the diagnosis of oral diseases.^2^ Dental radiographs whether it is for a single tooth or for the whole mouth along with the supporting structures have always assisted the dentist in assessing the oral health of a particular patient and providing adequate treatment for the dental condition. Therefore installing an x-ray unit is foremost requirement of a dentist before starting with dental set up.^4^

The dental radiographs in terms of radiation dosage are low and are said to cause minimal radiation hazard to the patient as well as dentist and the radiographer. According to the data, out of the total radiographs done, around 15% of them are dental radiographs and around 480 million radiographs are done on a yearly basis for the diagnosis of dental conditions.^5^ Even if the radiation hazard posed by the dental radiographs is low but still accumulation of such low level radiation in human body over a time period can pose a threat to the overall health of the individual. These concerns regarding the accumulation of radiation in human body can be due to inadvertent use of radiation to take x-rays, use of x-ray machines which have become older leading to leakage of x-rays and more x-rays, invention of cone beam computed tomography and inadequate knowledge of x-rays and their side effects among dental students, dentists, radiographers and other health care workers involved in taking the x-rays.^6^

The diagnosis of an oral condition by a dentist is done by taking radiographs of their patients. These dentists are trained and given adequate knowledge about taking radiographs with minimal health hazards at various levels of under graduation as well as post graduation. Clinical training is given during their clinical rotation whereas theoretical knowledge is imparted via lectures.^5^

Undergraduate students are allowed to take dental radiographs in Nepal under the supervision of trained and specialist dentist in the particular field. In Nepal, the undergraduate training regarding this particular subject is divided into 3rd and 4th year of the study of Bachelor of Dental Surgery (BDS) curriculum and during their one year compulsory rotatory internship. The students are trained and given adequate knowledge about the basics of radiology including physics of radiation, radiation biology and radiation hazards along with other advanced topics. The students also undergo clinical training in the department of Oral Medicine and Radiology in their 3rd and 4th year of BDS course.^7^

The concept of “As Low as Reasonably Achievable” (ALARA) should be implicated in clinical oral radiology which suggests that radiation should be as low which can be achieved within practical limits. There should be a balance between the side effects and the benefits produced by a single radiograph. Speaking in detail that every radiograph should only be done if it increases the ability of a dentist to diagnose a condition and treat it by comparing them with the side effects it is going to have on the patient. If there is a net benefit to the patient, only then the radiograph should be done. The BDS students should have proper knowledge of this concept so that they can provide the maximum benefit to the patient along with taking care of the health of their staff and other individuals sitting in their dental set up.^8^

After the student has completed his/her graduation, he/she is expected to take the dental radiographs with great precision, minimum attempts showing fine details following all the concepts of image formation along with protection of himself, his staff and patients from radiation hazards as far as possible. These may include wearing of lead apron and thyroid collar, following position distance rule and all the safety requirements of x-ray room. Ultimately, these undergraduate budding dentists would finally bloom into a well established dentist who will perform the dental radiographs at their own private practice. Therefore, it is very important to assess the knowledge of these undergraduate students regarding the dental radiographs and hazards caused by the radiation.^9^

The concept of ALARA is very important when radiology comes into picture which suggests that radiation should be as low which can be achieved within practical limits.^8^ There should be a balance between the side effects and the benefits produced by a single radiograph. Speaking in detail that every radiograph should only be done if it increases the ability of a dentist to diagnose a condition and treat it by comparing them with the side effects it is going to have on the patient. If there is a net benefit to the patient, only then the radiograph should be done.

The dental students should have proper knowledge of this concept so that they can provide the maximum benefit to the patient along with taking care of the health of their staff and other individuals sitting in their dental set up.^10^

Majority of the students that is, 85% enrolled in the present study had good knowledge about the radiation physics, protection and hazards. They also had knowledge about the implication of theoretical knowledge to clinical area so as to set up a radiation hazard free clinical practice. This study was in correlation with a previous study done by Swapna et al 2017 which also produced the similar results when it came to the knowledge assessment of dental students.^2^ However the results were not similar to a study conducted by Enabulele et al 2015 in which only 50% of students were familiar with the hazards of radiation.^3^ But still most of them scored in between 5–7 which are good according to the parameters taken but average in general.

The health hazards of x-rays to human beings which include genetic mutations, blood cancers and oncogenesis are very common due to the unawareness of these effects from radiation. Along with these man-made sources of radiation, the human beings are also exposed to radiation from natural sources and these radiations cause the death of 100–250 individuals in United Kingdom over one year.^10^ These deaths can be attributed to the poor knowledge about radiation safety and hazards among dental students, medical students, dentists, health care workers and radiographers. This suggests that the dental students and dentists should be armed adequate knowledge and training about radiation safety and hazards along with their safe practice. But unfortunately, the knowledge about this topic among dental students and dentists is lacking which is suggested by the inappropriate radiation protection practice by them in the dental set up.^11^

The results of present study suggest that although the majority of students had good knowledge about the radiation physics, hazards and protection, still most of the majority fell into the average knowledge category. More and more efforts should be incorporated in providing adequate knowledge and training both theoretically as well as clinically so that these budding dentists can do justice with the humanity by weighing the benefits of doing x-rays over the hazards of it. One should be able to apply ALARA concept every time and save the humanity from the hazards of radiation.

## CONCLUSIONS

The study concluded that even though the majority of students had good knowledge about the radiation physics, hazards and protection; most of the majority still fell into the average knowledge category. So, the teachers should involve in imparting more and more knowledge as well as training about the topic and BDS curriculum should be altered so as to train the budding dentists about all the aspects of radiation.

## Conflict of Interest

**None.**
